# An observational pilot evaluation of the Walk with Ease program for reducing fall risk among older adults

**DOI:** 10.1186/s13690-023-01219-8

**Published:** 2023-11-20

**Authors:** Nicholas R. Lamoureux, Jeni Lansing, Gregory J. Welk

**Affiliations:** 1 Department of Kinesiology and Sport Sciences, University of Nebraska at Kearney, Cushing Coliseum W220, 1410W 26th St, Kearney, NE 68849 USA; 2 Department of Kinesiology and Health, Iowa State University, Ames, IA USA

**Keywords:** Physical activity, Fall prevention, Healthy aging

## Abstract

**Background:**

Physical activity is an effective method of reducing fall risk among older adults. Previous evaluations of the six-week Walk with Ease (WWE) program have documented benefits to functional outcomes, but the potential effects on reducing fall risk have not been evaluated. This pilot study evaluates outcomes of a community delivered WWE program for potential suitability as a fall risk reduction program.

**Methods:**

A total of 59 older adults (age > 60) enrolled in a group version of WWE delivered by trained community-based leaders. Complete data (pre- and post-program) from functional fitness tests and behavioral instruments were obtained from 41 participants (aged 74.4 ± 6.6 years, 70% female). Functional outcomes included the 10-foot timed up and go (TUG), 30-second chair stand (CST) and 4-stage balance test (BT) included as part of STEADI, as well as a two-minute step test (ST) and normal gait speed test (GST). Survey assessments included STEADI fall risk screening, self-reported physical activity, and fear of falling measures. Analyses focused on reporting pre-post effect sizes, but paired t-tests were used to test statistical significance of differences.

**Results:**

Improvements in functional performance approached significance for both CST (d = 0.31, p = 0.06) and ST (d = 0.26, p = 0.12), but all other tests were nonsignificant. Survey results demonstrated significant increases in self-reported walking (d = 0.54, p = 0.02) and moderate-to-vigorous physical activity (MVPA; d = 0.56, p = 0.004), but perceived fear of falling and overall fall risk scores had smaller, non-significant, effects (d ranging from 0.01 to 0.31). Stratified analysis suggested that participants screened at an elevated risk for falls at baseline consistently had larger effects on all functional and survey assessments, though the analysis was underpowered to test significance.

**Conclusions:**

Walk with Ease participation significantly increased self-reported physical activity but did not significantly improve physical function or reduce fall risk. However, consistently larger effect sizes among participants screened as at-risk for falls suggest that the program may be beneficial for those with elevated risk for falls or functional limitations. Further research is needed to document the consistency of these effects among participants with elevated fall risk status.

**Table:** 

**Text box 1. Contributions to the literature**
➢ Participation in Walk with Ease may improve function and reduce risk of falling among individuals screened to be at higher risk for falling or those in early stages of functional decline.
➢ Participation in Walk with Ease can increase physical activity behavior in inactive older adults, potentially enabling the program to serve as a primary prevention strategy to prevent functional declines and decrease risk of falling in the future.
➢ These findings hsighlight the potential for community-based fall prevention programming that emphasizes a public health benefit through reach, acceptability, and sustainability.

## Background

Regular physical activity (PA) is a key preventative lifestyle behavior for individuals of all ages, but it is particularly important for older adults to support healthy aging due to its numerous benefits. In addition to well established reductions in mortality and chronic disease risk [1], physical activity also plays an important role in helping maintain function and independence, as well as reduce the risk for falls [2, 3]. One in four Americans over the age of 65 falls in a given year with significant consequences, as falls are the leading cause of injury related death among older adults [4, 5]. Additionally, approximately half of all community dwelling older adults demonstrate a fear of falling (FOF) [6]. For these reasons, fall prevention has been identified as an important priority both nationally within the United States [7], and internationally [8].

Evidence documents that physical activity is associated with improvements in several domains of physical function, helping to reduce both FOF [6, 9] and fall risk [10], while also helping to maintain independence and quality of life [3, 11]. Balance training and activities such as Tai Chi are frequently recommended for fall risk prevention, however, the need to train qualified instructors creates barriers for widespread implementation of these programs and limits accessibility [12]. Primary prevention strategies also warrant a focus on maintaining involvement in physical activity to reduce the need for more intensive fall prevention programming.

An alternative approach for scalable fall prevention efforts is through the promotion of basic lifestyle activities such as walking, which are widely accessible, and have been linked to reduced fall risk [13–16]. Some evidence has shown walking to be potentially more effective than balance training at preventing falls among low-risk older adults [17]. However delivery under less controlled conditions by non-expert program leaders may reduce intervention effectiveness in community settings with a wider range of participant function. Thus, further research is needed to test the potential of standard walking programs to improve functional fitness and reduce fall risk under conditions that are more reflective of real-world delivery.

A particularly promising program for widespread implementation is the Walk with Ease (WWE) program developed by the Arthritis Foundation. WWE is a structured, group-exercise program that emphasizes regular, self-paced walking and flexibility/stretching exercises across 6 weeks (18 sessions). Accumulated evidence supports the efficacy of WWE for promoting physical activity and functional improvements among older adults with arthritis [18, 19] and it has been endorsed by the Centers for Disease Control and Prevention (CDC) as a ‘lifestyle management’ program for older adults. Subsequent work has documented the potential for implementation into both community [20] and workplace [21] settings, but studies to date have not evaluated potential as a fall prevention program.

The present study adopts a pragmatic research design [22] to evaluate WWE implementation in a community-based setting. Although these pragmatic designs often introduce additional sources of variability or bias, it is crucial that evaluations are reflective of likely “real-world” outcomes to better represent the potential value to participants, clinicians, or community-based organizations prior to widespread dissemination. While both self-directed and group-based versions of the program are available, the group program was selected in this study as it provides more structure and accountability to guide and support participants. Group-based programs may be particularly beneficial to older adults due to the added social aspect of participation. Social support has been identified as a key motivator for increasing physical activity among older adults [23, 24], and interactions and support from peers are important for sustaining engagement in activity and receiving the ongoing benefits of activity [25]. Therefore, the primary purpose of this pilot study was to evaluate the efficacy of a group-based WWE program to promote improvements in physical activity and physical function among community-dwelling older adults. The evaluation included outcomes relevant to both immediate fall risk (i.e., function and fall risk screening) and that may lead to fall risk in the future (i.e., physical activity and FOF) to evaluate the potential for future studies and controlled trials evaluating fall risk.

## Method

### Overview of the Walk with Ease intervention

The study was conducted as a formalized evaluation of the standard, group-based version of the WWE program. WWE is designed to run as a 6-week program with 3 sessions per week (18 total). To facilitate enrollment and management, the program was adapted to operate on a rolling enrollment basis, with participants added weekly. This allowed for participant interest to be translated into immediate participation rather than the prolonged delays that may exist in a more traditional cohort model. The individualized approach to walking allowed participants to self-select their own intensity and duration; however, participants walked in the same indoor space to facilitate social interactions and the group-based session structure.

Session leaders arrived approximately 10 min prior to the session start time to facilitate conversation and engagement with participants, as well as welcome and orient new members. Each session followed the 5-step walking pattern outlined in the WWE leader manual and participant guidebook including; a warmup and stretching routine, a 30-minute period for self-paced walking, a cooldown and second stretching period, and a closing opportunity for socialization. Warmups consisted of five minutes of marching in place, followed by approximately 10 min of stretching, emphasizing the hips, thighs, and calves through a combination of standing and chair-based stretches. During the 30 min of walking, participants walked laps around the community space at their own pace, stopping to rest or drink water as needed. At the completion of the walking period, participants returned to the stretching area and completed the same stretching routine as a cool-down activity. Participants who missed sessions were able to come back until the full 18 sessions were completed, and participants were welcome to continue attending beyond the 18-session program timeline as space allowed, as a way of promoting ongoing engagement and social support for current participants. Participants who missed more than six sessions within their six-week enrollment were asked to re-enroll at a later date. All other participants who completed the program were included in analysis to reflect the nature of real-world program implementation.

### Recruitment of participants

Recruitment strategies emphasized the use of clinical partnerships to promote referrals of individuals who would benefit from participation in fall prevention programming, but flyers and mailers were also used to promote visibility among older adults. Participants aged 60 years or older who reported they were able to stand for at least 10 min without any increasing pain were eligible to participate. No exclusion criteria based on participant baseline function, activity, or residential status were placed on participants to support the naturalistic evaluation. All participants indicated they were safe to begin walking for exercise by completing the Physical Activity Readiness Questionnaire (PAR-Q) or by obtaining signed approval from a health care provider who was aware of all study components prior to them beginning program procedures.

### Design and program delivery

A non-experimental, pre-post design was used to systematically evaluate changes in physical fitness and function resulting from the standard implementation of the WWE program. The lead researcher completed the formalized WWE Leader training from the Arthritis Foundation to establish the infrastructure and act as a resource for session leaders, but the actual programming was managed by community members (e.g., regional health employees or individuals within the community that expressed an interest in establishing a walking program) that completed the WWE Leader training and followed the standard walking session protocol. Researchers provided feedback to session leaders regarding standardized adaptations made necessary due to the transition to an ongoing enrollment model (e.g., recommendations on integrating new participants into the group with ongoing enrollment), but session content remained consistent with the procedures outlined by the Arthritis Foundation leader training. All participants were also provided with the WWE Participant Guidebook and were advised to review the guidebook throughout their participation. Consistent with the guidebook, participants were also encouraged to progress towards walking outside of the scheduled sessions, as well as completing the program’s recommended stretching and strengthening routine at home at least twice each week.

### Primary outcome measures

Primary outcome measures were based on established indicators recommended by the CDC for the evaluation of fall risk [26]. The STEADI fall prevention toolkit has been validated as a screening tool for identifying individuals at elevated risk for falls through a series of screening and physical function assessments [27], has been widely adopted in healthcare settings [28], and the physical assessments have been used for evaluating changes to fall risk over time [29]. The STEADI toolkit includes a brief questionnaire and three primary functional tests, the Four-Stage Balance Test (BT), the 10-foot Timed Up and Go (TUG), and the 30-second chair stand test (CST). A two-minute step test (ST), and normal gait speed test (GST), as well as report-based assessments of FOF and physical activity were also included to provide a more complete assessment of fall-related outcomes. Each of the included assessments is commonly used among older adults.

#### STEADI Questionnaire:

The standardized STEADI protocol includes a brief 12-question “Stay Independent” survey that assesses risks of falling. Individuals are indicated as being at an elevated risk for falls by receiving a risk score of four or greater, or by responding ‘yes’ to any of the three “Key Questions” (they feel unsteady when standing or walking, they are worried about falling, or they have fallen in the past year). The self-report questionnaire has been shown to predict fall risk independent of function testing [30], and both scoring methods have been shown to be feasible in clinical settings.

#### 4-Stage Balance Test (BT):

The BT assesses static balance by having participants progress through four increasingly challenging stance positions (feet together; staggered stance; tandem stance; single leg stance) for up to 10 s each. Individuals who are not able to complete the tandem stance stage are considered at an elevated risk for falls. The assessment has demonstrated robust correlations with other measures of static balance, while being quick and simple to administer [31].

#### Timed Up and Go (TUG):

The TUG test is a commonly used fall risk screening assessment that times how long a participant takes to rise from a standard armchair, walk 10 feet (3.05 m), turn 180^o^, return to the chair and sit down [32]. Individuals who take more than 12 s to complete the test are considered at an elevated risk for falling. The TUG test has been noted as one of the most suitable balance performance measures among community dwelling older adults [33].

#### 30-second Chair Stand Test (CST):

In the CST, participants are asked to stand from a standard height chair with their arms crossed across the chest as many times as possible in 30 s [34]. The number of completed repetitions is then compared to age and gender-matched standards to determine individuals at an elevated risk for falls. The use of sit-to-stand tests has been used in previous evaluations due to high reliability, and evidence supporting the utility for assessing changes to function and strength, and is predictive of fall risk [35, 36].

### Secondary outcome measures

#### Two-Minute Step Test (ST):

The ST requires participants to march in place for two-minute by lifting the knee to mid-thigh height with each step and is scored as the number of completed step cycles within the two-minute time limit. The test has demonstrated good test-retest reliability, and convergent validity with 1-mile (1609.34 m) walk performance [37]. The test is also sensitive to improvements in participant function in response to rehabilitation programs, making it suitable for evaluating functional improvements in response to exercise programming [38], and results have demonstrated that completing 50 or fewer steps was associated with significantly increased fall risk [39].

#### Gait Speed Test (GST):

In the GST, participants are asked to walk a 20-foot (6.10 m) course including 4-foot (1.22 m) acceleration and deceleration zones at their self-selected “normal” walking pace [40]. The average of two trials is used to determine normal walking speed. A slow walking speed (≤ 1.0 m/s) has been linked to an increased risk of falls among older adults [41, 42].

#### Fear of Falling:

Fear of falling was assessed using the Falls Efficacy Scale International (FES-I) [43]. As part of the FES-I participants are asked to rate their level of concern with completing a series of common activities of daily living. The measure has previously demonstrated high test-retest reliability and adequately assesses concerns with engaging in basic and demanding activities. It can be used to identify individuals with high fear of falling, and has been recommended for use in falls related research [43, 44].

#### Self-reported physical activity

was assessed using the International Physical Activity Questionnaire – Elderly (IPAQ-E). Participants report the number of days in the past week, and the typical number of minutes per day engaged in moderate to vigorous physical activity (MVPA), walking, and sedentary activities. These values can then be used to calculate the total weekly PA, as well as categorize individuals in accordance with the recommended physical activity guidelines. The IPAQ-E has been shown to have adequate sensitivity and specificity for evaluating physical activity among older adults, based on studies making comparisons with other criterion measures [45].

### Research data collection procedures

All procedures were approved by the Institutional Review Board of the local University and all participants provided informed consent prior to their participation. After indicating they were safe to begin exercise, participants completed a baseline assessment of their physical function and fall risk screening using the STEADI fall risk functional assessments [46], as well as survey measures of physical activity and FOF. All assessments were completed by student research assistants trained to administer the assessments through a 2 credit practicum course. The participants were assessed by a single research assistant at baseline and post-program; however, transitions in student availability led to multiple students being involved over time. Inter-rater reliability assessments were not conducted during the study but subsequent work has demonstrated statistical equivalence between subjective ratings from student technicians and objective data from a customized biomechanical sensor (manuscript in development).

The functional assessments were conducted no more than one week prior to beginning the program and within one week of program completion. Participants were given an opportunity to rest as long as they felt was necessary between tests, and tests were sequenced to minimize the impact of fatigue (i.e., health status, BT, GST, TUG, CST, ST). After completing all assessments, participants were provided with information about the scheduling of the program and instructed to join the first session of the week following the completion of their assessments.

### Analysis

The study followed established guidelines from the STEADI protocol for evaluating fall risk of participants. The STEADI screening survey was analyzed using both established scoring metrics (score based and key question based) to categorize fall risk. The categorization of baseline fall risk is typically used as an indicator of the function of the participants but, in the present study, was also used as a way to facilitate segmentation of participants into high and low risk for falls. Descriptive summaries of functional test scores and physical activity were reported to further characterize the sample at baseline.

In addition to evaluating overall fall risk, functional indicators, physical activity, and fear of falling were also evaluated individually to provide a thorough evaluation of potential benefits of WWE participation. Emphasis was placed on calculated effect sizes for the main outcomes of interest, but paired t-tests were also used to test if differences between baseline and post-program outcomes were statistically significant. Effect sizes were calculated as the difference between baseline and post-program divided by the standard deviation of the differences. Although an increase in raw outcome score at post-program may indicate either an improvement (such as CST or ST scores), or a decline in function (such as TUG or FES-I scores), effect sizes are reported as positive values if the change reflected a beneficial effect.

## Results

A total of 59 participants enrolled and completed baseline assessment procedures. Enrolled participants were predominantly White, non-Hispanic (91.5%), females (70%) between 63 and 89 years old (mean 74.4 ± 6.6 years). Post-participation assessments were completed by 41 participants, with no significant differences in demographics between completers and non-completers. See Table 1 for a complete description of participants.

**Table 1:** Demographics of participants enrolled in (and completing) the WWE evaluation observational pilot trial

Characteristic	Enrolled (n = 59)	Completed (n = 41)
Gender (% Female)	70%	70.1%
Age (mean ± SD years)	74.4 ± 6.6	74.2 ± 6.4
BMI (mean ± SD kg/m^2^)	29.9 ± 6.7	30.4 ± 7.0
% Obese (BMI ≥ 30 kg/m^2^)	42.4%	43.9%
Ethnicity		
White (%)	91.5%	97.6%
Asian (%)	1.7%	2.4%
Not Reported (%)	6.8%	0%
Education		
High School Graduate	47.5%	46.3%
College Graduate	16.9%	19.5%
Graduate Degree	30.5%	34.1%
Not Reported	5.1%	0%

The STEADI survey responses at baseline provide insights about the fall-related concerns of older adults in the sample falling. Approximately 34% of participants reported falling in the past year, 31% indicated a fear of falling, and 40% indicated that they felt unsteady when walking. Approximately one third of participants also indicated other functional limitations that elevate the risk for falls, with 32% of participants indicating a need to use their arms to stand from a chair and 32% indicating difficulty stepping up on to a curb. Using the STEADI risk stratification system, approximately 55% of the participants were classified as having an elevated risk of falling by either the score based (i.e., scored ≥ 4 points on the Stay Independent questionnaire) or by the key questions method.^1^

To further examine functional profiles of the participants, results were stratified by baseline STEADI risk classification. Comparisons of baseline STEADI functional assessment performance showed that CST repetitions were significantly lower among those flagged at an elevated risk for falls. Additionally, significant differences in GST and FOF were noted based on fall risk classification. The BT, TUG test and ST also all revealed differences in the expected direction (i.e., lower function among those screened to be at an elevated fall risk), but these values did not reach significance. Assessments of physical activity showed no differences in self-reported MVPA or walking time between the two risk classification groups. See Table 2 for a complete summary of outcomes at baseline.

**Table 2:** Descriptive statistics for pilot trial participant function and activity at baseline (stratified by STEADI screening questionnaire risk status)

	Elevated Fall Risk (n = 30)	Low fall risk(n = 24)	P Value
STEADI Functional Assessments			
4-Stage Balance Test (seconds)	34.3 ± 6.12	35.9 ± 6.6	0.362
Timed up-and-go (seconds)	10.2 ± 3.45	8.8 ± 2.35	0.096
30-second Chair Stand (repetitions)	9.1 ± 4.48	12 ± 2.53	0.004
Additional Assessments			
2-minute Step Test (steps)	68.1 ± 21.9	79.1 ± 25.6	0.105
Normal Gait Speed (m/s)	1.02 ± 0.25	1.16 ± 0.24	0.036
Fear of Falling	28.7 ± 8.97	19.5 ± 2.09	< 0.0001
Self-Reported Physical Activity			
Self-Reported MVPA (mins)	233.7 ± 336.7	103.4 ± 211.1	0.192
Self-Reported Walking (mins)	194.3 ± 371.1	193.4 ± 294.6	0.640

MVPA moderate-to-vigorous physical activity

Change scores for the functional tests were analyzed to provide specific insights into program outcomes. Among the 39 participants who completed post-program functional evaluations, improvements were noted for most outcomes (See Table 3). Effect sizes showed small improvements to CST and ST performance for both high risk and low risk participants, though none of the improvements reached statistical significance. Small,non-significant, improvements were evident on the TUG test and BT performance among elevated risk participants but values did not improve among those with low fall risk. Changes to FOF showed that (on average) participants at elevated fall risk had small reductions in FOF, while those at low fall risk had less substantial reductions. Average self-reported levels of physical activity changes increased for the total sample, with larger increases in self-reported MVPA among those with low fall risk. Interestingly, there was minimal change in reported MVPA for those at higher risk for falls, though this may be a product of the limited sample size and the lack of precision of self-report measures as 83% of low risk and 62% of elevated risk participants reported increases in MVPA. Similarly, 90% of low risk and 67% of elevated risk participants reported increases in their weekly walking. Table 3 contains complete details on outcome differences from baseline to post-program, stratified by participant baseline STEADI fall risk classification.

**Table 3:** Overall differences in WWE outcomes from baseline to post-program, and results stratified by baseline STEADI fall risk stratification

Test	Difference from Baseline	Effect Size	p-value
STEADI Functional Assessment Score Differences			
4-Stage Balance Test (seconds)	0.07 ± 5.8	0.01	0.936
Low fall risk (n = 18)	-0.83 ± 6.2	-0.13	0.574
Elevated Fall Risk (n = 21)	1.04 ± 5.6	0.19	0.401
Timed up-and-go (seconds)	-0.06 ± 1.5	0.04	0.791
Low fall risk (n = 18)	0.37 ± 1.3	-0.29	0.229
Elevated Fall Risk (n = 21)	-0.44 ± 1.6	0.27	0.243
30-second Chair Stand (repetitions)	0.78 ± 2.5	0.31	0.056
Low fall risk (n = 18)	0.61 ± 2.2	0.27	0.260
Elevated Fall Risk (n = 21)	1.00 ± 2.9	0.34	0.132
Additional Assessments			
2-minute Step Test (steps)	5.31 ± 20.5	0.26	0.119
Low fall risk (n = 18)	6.83 ± 25.4	0.27	0.269
Elevated Fall Risk (n = 20)	4.89 ± 15.1	0.32	0.188
Normal Gait Speed (m/s)	0.01 ± 0.13	0.09	0.531
Low fall risk (n = 18)	-0.001 ± 0.13	-0.01	0.967
Elevated Fall Risk (n = 21)	0.02 ± 0.14	0.18	0.407
Fear of Falling	-1.2 ± 5.9	0.13	0.240
Low fall risk (n = 17)	0.31 ± 5.7	-0.06	0.828
Elevated Fall Risk (n = 22)	-2.72 ± 6.1	0.27	0.232
Physical Activity			
Self-Reported MVPA (mins)	294.8 ± 478.3	0.56	0.004
Low fall risk (n = 12)	527.5 ± 457.0	1.15	0.002
Elevated Fall Risk (n = 14)	50.76 ± 391.9	0.04	0.858
Self-Reported Walking (mins)	243.2 ± 450.9	0.54	0.020
Low fall risk (n = 10)	286.50 ± 431.8	0.66	0.065
Elevated Fall Risk (n = 12)	207.08 ± 482.1	0.43	0.165

*Note*: Change in test differences from baseline reflects the change in raw data collected and may represent an improvement or decrement in performance. Effect sizes are reported with positive effect indicating an improvement in performance. Continuous Fall Risk Score changes are reported after removal of 4 outlier participants. MVPA: Moderate-to-Vigorous Physical Activity

The aggregated results obscure some of the variability in responses noted at the individual level. For example, 10 of 40 participants (25%) who completed the TUG test at both time points improved by at least 0.8 s, and 11 of 41 participants (27%) improved by at least 2 repetitions during CST, both of which have been associated with important improvements in function [47]. Despite these clinically significant improvements, nearly all participants retained their baseline risk classification.

Additionally, while normal gait speed is not included in STEADI, gait speeds slower than 1.0 m per second are associated with elevated fall risk [48], and improvements to gait speed have been shown to predict eight-year survival rates among older adults [49]. In the present study, three participants had improvements that moved them above this 1 m/s threshold [50, 51], and 21 (51%) had increases in gait speed. The improvements in overall underlying function suggest that the program may provide meaningful benefits for some participants even if changes in functional risk indicators don’t change.

## Discussion

The main purpose of this study was to examine changes in physical activity, functional fitness and fall risk indicators following implementation of WWE in older adults. The program led to moderate to large increases in self-reported physical activity (ES = 0.54 to 0.56) but small changes in functional fitness indicators (ES ranging from 0.01 to 0.31) and fear of falling (ES = 0.13). While the overall effects are small, the patterns reflect important gains in functional fitness and suggest possible benefits for fall prevention.

The study was not designed to evaluate moderating influences; however, sub-analyses revealed participants screened at elevated risk for falls at baseline consistently had larger improvements in all functional tests, and greater reductions in FOF. Thus, the use of a heterogenous sample of community-dwelling adults may have muted the overall findings on the impact of the program on function and fall risk. It should also be noted that the elevated risk participants were still relatively high functioning based on the functional assessment scores, likely in the early stages of decreasing function, and further research into which population subgroups would optimally benefit from participation in WWE is necessary.

Previous evaluations of WWE have also noted the ability to improve physical function, with Callahan et al. [19] demonstrating that WWE contributed to improvements in one and three repetition chair stand time, 360^o^ turn and single leg stance time, and walking speed. This study extends this work by demonstrating the potential for improvements to function, as well as the potential for mitigating fall risk and FOF, particularly among individuals with an existing elevated risk for falls. Notably, previous research did not show improvements in ST performance, while the present study showed small improvements. It is not clear how to explain these differences, but the present results are in line with other evaluations of walking-based interventions, which have shown improvements in aerobic capacity with significant individual variability in effects [52, 53]. Consistently larger effect sizes on functional assessments among those with elevated baseline fall risk suggests that the program, as currently delivered, may have benefits for individuals with declining physical function. Those with higher function may not see the same improvements, though the noted large increases in physical activity may help in preventing functional decline. Thus, the program may have benefits as a primary prevention strategy.

Evidence supporting walking-based fall prevention interventions already exists, highlighted by Okubo et al. [17] who showed advantages relative to standard balance-based programs. The brisk walking intervention produced similar overall reductions in the incidence of falls as balance training and lower fall rates when standardized to exposures (i.e., days that contained physical activity or steps taken). The authors suggested that the brisk walking helped to improve endurance and reduce falls related to fatigue, while also exerting a potential inoculating effect of “involuntary stepping training”, improving recovery from minor trips to prevent falls. Participants in the walking group had higher frequencies of trips (defined as stumbling over an object without landing on any part of the body), which may make brisk walking interventions inappropriate for high fall risk individuals due to functional deficits. The WWE program includes walking and stretching/strengthening exercise so it is not possible to determine the relative contributions of these forms of exercise to the improvements noted in the study.

The gains reported in the study are important considering that the program lasted only 6 weeks. While significant improvements were not observed in participant function, there were significant, moderate increases in self-reported activity. If participants are able to maintain increased physical activity behaviors, it may also translate to greater functional improvements over time. Although Callahan et al. [19] did not include an evaluation of physical activity behavior, other evaluations have shown that WWE participation is linked with increased PA. Nyrop at al [18]. concluded that participants in both group and self-directed versions of the program reported increased weekly walking, and continued walking at a one-year follow up, and Conte et al. [20] demonstrated that participants reported additional days of walking each week. It should also be noted that due to a lack of measure precision inherent to self-reported physical activity data, the changes in the present study reflect only overall group level effects, rather than expected individual changes to physical activity. While the estimates should be treated with caution, the majority of participants reported weekly increases in both MVPA and walking which can help improve function long term, positively impacting fall risk. A more robust assessment of individual PA changes would require widespread use of device-based assessments to increase measure reliability and was outside the scope of this evaluation.

Strengths of the study include the use of an existing evidence-based program that is widely available and a naturalistic design that captures changes in functional fitness outcomes from community-dwelling adults under real-world conditions. The community-based implementation yielded small effects for the aggregated sample but considerable heterogeneity in outcomes were observed at the individual level. Variability in outcomes is common in translational research as it is difficult to replicate outcomes from controlled efficacy studies when delivered in real-world settings [54, 55]. Future efforts to supplement the existing WWE program with additional home-based strengthening exercises may lead to stronger outcomes, helping to maximize the population impact of the broadly disseminated program.

There are clear limitations associated with non-experimental designs, but the goal of the pilot study was to evaluate typical changes in older adults enrolling in WWE to determine the suitability for further study, rather than to evaluate comparative efficacy or determine the source of beneficial outcomes. Emphasis was also placed on a naturalistic design by evaluating a community-based implementation rather than delivery from researchers or other experts to ensure findings are reflective of real-world delivery. However, the predominantly female, white non-Hispanic sample, makes it difficult to generalize the present findings to other, more representative populations. Predominantly female samples are an established challenge among older adult physical activity research [56], and ongoing efforts to engage and retain older adult male participants remain a priority. Additionally, while a quarter to half of all participants noted improvements on a given functional assessment, few participants were able to improve their physical function enough to change their STEADI fall risk classification, and it remains unclear if WWE participation meaningfully reduces fall risk, and which participants would optimally benefit from participation. Further research with long-term follow-up is necessary to determine if the lack of risk status change is (a) due to the functional improvements not significantly reducing fall risk or if it (b) represents an inability of the STEADI classification to detect improvements among those with elevated fall risk. Individuals may improve their function, but it may not be detectable by current fall screening methods without corresponding reductions in FOF, which is a key indicator in both scoring versions of STEADI.

Follow-up trials are needed to document the long-term effects of the program on fall incidence, as well as to identify the individual risk factors that most benefit from participation. An advantage of the WWE program is the emphasis on accessible, self-guided walking which has been linked to increased participant adherence over time [57]. WWE may prove to have a greater population-level impact than more complex programs due to its already widespread adoption. Future research may also begin evaluating potential improvements to various components of WWE, such as alternative home-based muscular strengthening exercise prescriptions that further improve functional outcomes.

## Conclusions

The WWE program may help promote physical activity and improve physical function in older adults at an elevated risk for falls – potentially helping lower long-term risk of falling. Further research is needed to identify the population subgroups that optimally benefit, but preliminary results suggest that it may be a suitable program for reducing risk among relatively high functioning individuals that have been screened at an elevated risk for falls. Iterative cycles of improvement to increase the magnitude of individual outcome benefits of WWE holds to potential for meaningful population benefits due to its widespread adoption and broad reach, allowing many community-based organizations to offer the program within their existing capacity.

## Data Availability

The dataset used during the current study is available from the corresponding author on reasonable request.

## Footnotes

1 Comparisons were made between the risk assessments from the two different STEADI classification approaches. The majority of participants (73%) were classified similarly with both methods; however, to streamline interpretations, participants were flagged as at risk if they met either criteria.

## List of abbreviations

CDC: Centers for Disease Control and Prevention
CST: Chair stand test
FOF: Fear of falling
BT: Four stage balance test
GST: Gait Speed Test
STEADI: Stopping Elderly Accidents, Deaths, and Injuries
TUG: Timed Up-and-Go
ST: Two-minute step test
WWE: Walk with Ease

## References

[1] Warburton DE, Bredin SS (2017). Health benefits of physical activity: a systematic review of current systematic reviews. Curr Opin Cardiol.

[2] Paterson DH, Warburton DE (2010). Physical activity and functional limitations in older adults: a systematic review related to Canada’s Physical Activity guidelines. Int J Behav Nutr Phys Activity.

[3] Tak E, Kuiper R, Chorus A, Hopman-Rock M (2013). Prevention of onset and progression of basic ADL disability by physical activity in community dwelling older adults: a meta-analysis. Ageing Res Rev.

[4] Kulinski K, DiCocco C, Skowronski S, Sprowls P (2017). Advancing community-based falls prevention programs for older adults—the work of the Administration for Community Living/Administration on Aging. Front Public Health.

[5] Stevens J, Ballesteros MF, Mack KA, Rudd RA, DeCaro E, Adler G (2012). Gender differences in seeking care for falls in the aged Medicare population. Am J Prev Med.

[6] Zijlstra GR, Van Haastregt JC, Van Rossum E, Van Eijk JTM, Yardley L, Kempen GI (2007). Interventions to reduce fear of falling in community-living older people: a systematic review. J Am Geriatr Soc.

[7] US Department of Health and Human Services. Healthy People 2030 - Physical Activity Workgroup 2021 [Available from: https://health.gov/healthypeople/about/workgroups/physical-activity-workgroup.

[8] World Health Organization. Decade of healthy ageing: baseline report. 2020.

[9] Kumar A, Delbaere K, Zijlstra G, Carpenter H, Iliffe S, Masud T (2016). Exercise for reducing fear of falling in older people living in the community: Cochrane systematic review and meta-analysis. Age Ageing.

[10] Manini TM, Pahor M (2009). Physical activity and maintaining physical function in older adults. Br J Sports Med.

[11] Chase J-AD, Phillips LJ, Brown M (2017). Physical activity intervention effects on physical function among community-dwelling older adults: a systematic review and meta-analysis. J Aging Phys Act.

[12] Ory MG, Smith ML, Parker EM, Jiang L, Chen S, Wilson AD (2015). Fall prevention in community settings: results from implementing Tai Chi: moving for Better Balance in three states. Front Public Health.

[13] McMullan II, McDonough SM, Tully MA, Cupples M, Casson K, Bunting BP (2018). The association between balance and free-living physical activity in an older community-dwelling adult population: a systematic review and meta-analysis. BMC Public Health.

[14] Battaglia G, Giustino V, Messina G, Faraone M, Brusa J, Bordonali A (2020). Walking in natural environments as geriatrician’s recommendation for fall prevention: preliminary outcomes from the passiata day model. Sustainability.

[15] McPhee JS, French DP, Jackson D, Nazroo J, Pendleton N, Degens H (2016). Physical activity in older age: perspectives for healthy ageing and frailty. Biogerontology.

[16] Sherrington C, Tiedemann A, Fairhall N, Close JC, Lord SR (2011). Exercise to prevent falls in older adults: an updated meta-analysis and best practice recommendations. N S W Public Health Bull.

[17] Okubo Y, Osuka Y, Jung S, Rafael F, Tsujimoto T, Aiba T (2016). Walking can be more effective than balance training in fall prevention among community-dwelling older adults. Geriatr Gerontol Int.

[18] Nyrop KA, Cleveland R, Callahan LF (2014). Achievement of exercise objectives and satisfaction with the Walk with ease program—group and self-directed participants. Am J Health Promotion.

[19] Callahan L, Shreffler JH, Altpeter M, Schoster B, Hootman J, Houenou LO (2011). Evaluation of group and self-directed formats of the Arthritis Foundation’s Walk with Ease Program. Arthritis Care Res.

[20] Conte KP, Odden MC, Linton NM, Harvey SM (2016). Effectiveness of a scaled-up arthritis self-management program in Oregon: walk with ease. Am J Public Health.

[21] Silverstein RP, VanderVos M, Welch H, Long A, Kaboré CD, Hootman JM (2018). Self-Directed Walk with Ease Workplace Wellness Program—Montana, 2015–2017. Morb Mortal Wkly Rep.

[22] Porzsolt F, Rocha NG, Toledo-Arruda AC, Thomaz TG, Moraes C, Bessa-Guerra TR (2015). Efficacy and effectiveness trials have different goals, use different tools, and generate different messages. Pragmatic and Observational Research.

[23] Costello E, Kafchinski M, Vrazel J, Sullivan P (2011). Motivators, barriers, and beliefs regarding physical activity in an older adult population. J Geriatr Phys Ther.

[24] Smith GL, Banting L, Eime R, O’Sullivan G, Van Uffelen JG (2017). The association between social support and physical activity in older adults: a systematic review. Int J Behav Nutr Phys Activity.

[25] Franco MR, Tong A, Howard K, Sherrington C, Ferreira PH, Pinto RZ (2015). Older people’s perspectives on participation in physical activity: a systematic review and thematic synthesis of qualitative literature. Br J Sports Med.

[26] CDC. STEADI - Older Adult Fall Prevention: Centers for Disease Control and Prevention, National Center for Injury Prevention and Control. 2023. Available from: https://www.cdc.gov/steadi/index.html.

[27] Lohman MC, Crow RS, DiMilia PR, Nicklett EJ, Bruce ML, Batsis JA (2017). Operationalisation and validation of the stopping Elderly Accidents, deaths, and injuries (STEADI) fall risk algorithm in a nationally representative sample. J Epidemiol Community Health.

[28] Sarmiento K, Lee R, STEADI (2017). CDC’s approach to make older adult fall prevention part of every primary care practice. J Saf Res.

[29] Frith KH, Hunter AN, Coffey SS, Khan Z (2019). A longitudinal fall prevention study for older adults. J Nurse Practitioners.

[30] Ritchey K, Olney A, Chen S, Phelan EA (2022). STEADI self-report measures independently predict fall risk. Gerontol Geriatric Med.

[31] Rossiter-Fornoff JE, Wolf SL, Wolfson LI, Buchner DM, Group F (1995). A cross-sectional validation study of the FICSIT common data base static balance measures. The Journals of Gerontology Series A: Biological Sciences and Medical Sciences.

[32] Podsiadlo D, Richardson S (1991). The timed up & go: a test of basic functional mobility for frail elderly persons. J Am Geriatr Soc.

[33] Rydwik E, Bergland A, Forsén L, Frändin K (2011). Psychometric properties of timed up and go in elderly people: a systematic review. Phys Occup Therapy Geriatr.

[34] Jones CJ, Rikli RE, Beam WC (1999). A 30-s chair-stand test as a measure of lower body strength in community-residing older adults. Res Q Exerc Sport.

[35] Goldberg A, Chavis M, Watkins J, Wilson T (2012). The five-times-sit-to-stand test: validity, reliability and detectable change in older females. Aging Clin Exp Res.

[36] Trommelen RD, Buttone LF, Dicharry DZ, Jacobs RM, Karpinski A (2015). The use of five repetition sit to stand test (FRSTST) to assess fall risk in the assisted living population. Phys Occup Therapy Geriatr.

[37] Rikli RE, Jones CJ (1999). Development and validation of a functional fitness test for community-residing older adults. J Aging Phys Act.

[38] Haas F, Sweeney G, Pierre A, Plusch T, Whiteson J (2017). Validation of a 2 minute step test for assessing functional improvement. Open J Therapy Rehabilitation.

[39] Toraman A, Yıldırım NÜ (2010). The falling risk and physical fitness in older people. Arch Gerontol Geriatr.

[40] Fritz S, Lusardi M (2009). White paper:walking speed: the sixth vital sign. J Geriatr Phys Ther.

[41] Quach L, Galica AM, Jones RN, Procter-Gray E, Manor B, Hannan MT (2011). The nonlinear relationship between gait speed and falls: the maintenance of balance, Independent living, intellect, and zest in the elderly of Boston study. J Am Geriatr Soc.

[42] Kyrdalen IL, Thingstad P, Sandvik L, Ormstad H (2019). Associations between gait speed and well-known fall risk factors among community‐dwelling older adults. Physiotherapy Res Int.

[43] Yardley L, Beyer N, Hauer K, Kempen G, Piot-Ziegler C, Todd C (2005). Development and initial validation of the Falls Efficacy Scale-International (FES-I). Age Ageing.

[44] Delbaere K, Close JC, Mikolaizak AS, Sachdev PS, Brodaty H, Lord SR (2010). The falls efficacy scale international (FES-I). A comprehensive longitudinal validation study. Age Ageing.

[45] Hurtig-Wennlöf A, Hagströmer M, Olsson LA (2010). The International Physical Activity Questionnaire modified for the elderly: aspects of validity and feasibility. Public Health Nutr.

[46] Stevens J (2013). The STEADI tool kit: a fall prevention resource for health care providers. IHS Prim care Provider.

[47] Dobson F, Hinman RS, Hall M, Terwee C, Roos EM, Bennell K (2012). Measurement properties of performance-based measures to assess physical function in hip and knee osteoarthritis: a systematic review. Osteoarthr Cartil.

[48] Espy DD, Yang F, Bhatt T, Pai Y-C (2010). Independent influence of gait speed and step length on stability and fall risk. Gait Posture.

[49] Hardy SE, Perera S, Roumani YF, Chandler JM, Studenski SA (2007). Improvement in usual gait speed predicts better survival in older adults. J Am Geriatr Soc.

[50] Pamoukdjian F, Paillaud E, Zelek L, Laurent M, Levy V, Landre T (2015). Measurement of gait speed in older adults to identify Complications associated with frailty: a systematic review. J Geriatric Oncol.

[51] Verghese J, Wang C, Holtzer R (2011). Relationship of clinic-based gait speed measurement to limitations in community-based activities in older adults. Arch Phys Med Rehabil.

[52] Murphy MH, Nevill AM, Murtagh EM, Holder RL (2007). The effect of walking on fitness, fatness and resting blood pressure: a meta-analysis of randomised, controlled trials. Prev Med.

[53] Whipple MO, Schorr EN, Talley KM, Lindquist R, Bronas UG, Treat-Jacobson D (2018). Variability in individual response to aerobic exercise interventions among older adults. J Aging Phys Act.

[54] Durlak JA, DuPre EP (2008). Implementation matters: a review of research on the influence of implementation on program outcomes and the factors affecting implementation. Am J Community Psychol.

[55] Giné-Garriga M, Roqué-Fíguls M, Coll-Planas L, Sitja-Rabert M, Salvà A (2014). Physical exercise interventions for improving performance-based measures of physical function in community-dwelling, frail older adults: a systematic review and meta-analysis. Arch Phys Med Rehabil.

[56] Chase J-AD (2013). Physical activity interventions among older adults: a literature review. Res Theory Nurs Pract.

[57] Hillsdon M, Thorogood M (1996). A systematic review of physical activity promotion strategies. Br J Sports Med.

